# CircCSPP1 Competitively Binds miR-10a to Regulate BMP7 Expression and Affects the Proliferation of Dermal Papilla Cells

**DOI:** 10.3390/ijms252111547

**Published:** 2024-10-27

**Authors:** Xiaoyang Lv, Jie Wang, Yeling Xu, Hui Zhou, Yutao Li, Wei Sun

**Affiliations:** 1 Joint International Research Laboratory of Agriculture and Agri-Product Safety of Ministry of Education of China, Yangzhou University, Yangzhou 225009, China; dx120170085@yzu.edu.cn; 2 International Joint Research Laboratory in Universities of Jiangsu Province of China for Domestic Animal Germplasm Resources and Genetic Improvement, Yangzhou University, Yangzhou 225009, China; 3 College of Animal Science and Technology, Yangzhou University, Yangzhou 225009, China; 4 CSIRO Agriculture and Food, 306 Carmody Rd., St. Lucia, QLD 4067, Australia

**Keywords:** circCSPP1, miR-10a, BMP7, dermal papilla cell, proliferation

## Abstract

A series of differentially expressed circular RNAs (circRNAs), microRNAs (miRNAs), and messenger RNAs (mRNAs) were identified through sequencing in the hair follicle tissues of Hu sheep with small-waved and straight wool patterns. Based on these findings, the circCSPP1-miR-10a-*BMP7* (Bone Morphogenetic Protein 7) regulatory network was constructed. The preliminary study highlighted that miR-10a and the BMP7 gene exhibited not only significant differential expression across hair follicle tissues with different patterns in Hu sheep but also had an impact on the proliferation of hair papilla cells. The proliferation of hair papilla cells is intricately linked to hair follicle development and growth. Consequently, we selected the circCSPP1-miR-10a-*BMP7* regulatory network to validate its role in promoting hair papilla cell proliferation in Hu sheep. Firstly, the authenticity of circCSPP1 was successfully confirmed through RNase R digestion and reverse primer amplification. Additionally, nucleoplasmic localization analysis determined that circCSPP1 was predominantly distributed in the cytoplasm. Using the dual-luciferase gene reporter system, we verified the targeting relationship between circCSPP1 and miR-10a, building upon our previous validation of the miR-10a-*BMP7* interaction. This clarified the competing endogenous RNA (ceRNA) mechanism within the circCSPP1-miR-10a-*BMP7*. Furthermore, rescue experiments confirmed that circCSPP1 competitively binds to miR-10a, thereby regulating *BMP7* expression and influencing the proliferation of hair papilla cells in Hu sheep. This discovery provides a solid foundation for future investigations into the mechanisms underlying wool curvature and the formation of lambskin patterns, offering insights into the complex regulatory networks that govern these phenotypic traits in Hu sheep.

## 1. Introduction

The Hu sheep, a unique breed with white lambskin originating from China, has lots of excellent qualities that have earned it a prominent position within the country’s domestic sheep germplasm genetic resources. It possess significant conservation and reserve value. The lambskin of Hu sheep, renowned for its distinct wavy pattern, can be categorized into four types: large wave, medium wave, small wave, and straight wool. Among these, the type of pattern serves as a pivotal factor in assessing the quality of the lambskin, with the small wave pattern universally acknowledged as the superior quality, whereas the straight wool is typically considered the least desirable. However, in recent years, the rapid expansion of the meat sheep market has prioritized breeding practices focused on enhancing meat qualities, inadvertently neglecting the unique lambskin traits of Hu sheep, leading to a decline in the quality of their lambskins. The precious germplasm resources of purebred Hu sheep, renowned for their exceptional lambskin patterns, are now under grave threat. Therefore, it is urgently needed to carry out a study of the formation of the molecular mechanisms. Wool formation is closely linked to the growth and development of the hair follicle; the growth of the wool fibre starts from hair follicles in the skin and the growth and development of the hair follicle interacts with various signalling pathways to form a complex regulatory network that affects the function of different cell types in the hair follicle structure, resulting in the curling of the wool of the lambs to form a wavy pattern. The dermal papilla cells (DPCs) are located at the base of the hair follicle and are encapsulated by the hair matrix cells. The DPCs act as a signalling centre playing an important role in hair matrix cells differentiation and hair growth [1,2].

Bone morphogenetic proteins (BMPs) are a class of structurally similar and highly conserved functional proteins, and BMP signalling is important in controlling cell differentiation and apoptosis during hair follicle development, as well as for regulating key steps in hair follicle development [3]. With the deepening of research, more and more BMP family genes have been discovered in the hair follicle, such as *BMP2*, *BMP4*, and *BMP7*, which are expressed in the hair follicle [4]. Bin et al. [5], investigating the role of DPCs in wound healing, found that *BMP7* counteracted the effect of *TGF-β1* in inducing DPCs’ differentiation into fibroblasts. Esibizione et al. [6] also demonstrated that *BMP7* is expressed in the hair follicle and that its expression is altered during hair follicle development in mice. The group’s previous research found that the *BMP7* gene is associated with indicators of hair follicle development [7] and can directly affect the proliferation of hair papilla cells in Hu lambs [8].

CircRNAs are found mainly in the cytoplasm, have the characteristics of high stability, tissue specificity, and temporal sequence specificity, and play a role in a variety of biological processes. At present, research on circRNAs is mainly focused on diseases, and there are not many applications in livestock and very few studies on the growth and development of hair follicle. The study of circRNAs in the hair follicle is still at the stage of circRNA identification and preliminary network construction, and in-depth regulatory mechanisms are still rare. Zhao et al. [9] identified 8753 circRNAs in the hair follicles of Aohan Merino sheep. Zhao et al. [10] performed RNA-seq on the various stages of the hair follicle cycle in rabbits, identified 247 differentially expressed circRNAs, and constructed a circRNA-miRNA-mRNA network. In addition, in a previous study, 114 differentially expressed circRNAs were screened via RNA-seq in the straight and small wave wool of Hu sheep, a circRNA-miRNA regulatory network was constructed, and circCSPP1-miR-10a was discovered [11]. MicroRNAs (miRNAs) are highly conserved non-coding RNAs of approximately 18–25 nt in length across species and are able to regulate gene expression at the post-transcriptional level by either inhibiting messenger RNA (mRNA) translation or promoting mRNA degradation [12,13]. miRNAs form a diverse regulatory network with their target genes and also play an important role in hair follicle growth and development. Previous studies have found that miR-10a is a differentially expressed miRNA between small wave and large wave wool in Hu sheep and has a targeting relationship with *BMP7* [14]. Furthermore, preliminary RNA-seq analysis revealed that circCSPP1 is a differentially expressed circRNA between the straight wool and small waves groups [11]. Additionally, a potential targeting relationship between circCSPP1 and miR-10a was predicted, leading us to select circCSPP1-miR-10a-*BMP7* as the focus of this research.

Competitive endogenous RNAs (ceRNAs) have been a hot topic of research in recent years. miRNAs can cause gene silencing by degrading mRNAs by binding to their 3‘UTR regions, while ceRNAs can regulate gene expression by competitively binding to miRNAs. ceRNAs, as a completely new mode of gene expression regulation, are more delicate and complex, involving more RNA molecules compared to the regulatory network between miRNAs and genes [15,16]. The ceRNA mechanism has been increasingly studied in domestic animals, particularly for traits such as muscle characteristics, reproductive traits, etc. Elnour et al. [17] found that circMYL1 expression was downregulated during myoblast proliferation and progressively upregulated during myoblast differentiation, and further demonstrated that circMYL1 could inhibit myoblast proliferation and promote differentiation by binding to miR-2400. Li et al. [18,19] showed in their study of bovine muscle development that circFGFR4 binding to miR-107 promotes cell differentiation of primary bovine myoblasts by targeting *Wnt3a* and that circFUT10 regulates myoblast differentiation by directly binding to miR-133a and inhibiting its activity.

In this study, we constructed the ceRNA regulatory network of circCSPP1-miR-10a-*BMP7* and investigated its effect on the proliferation of DPCs, which provides some basis for the subsequent study of hair follicle growth and development and pattern formation in Hu sheep.

## 2. Results

### 2.1. Identification of circCSPP1

**Figure 1:**
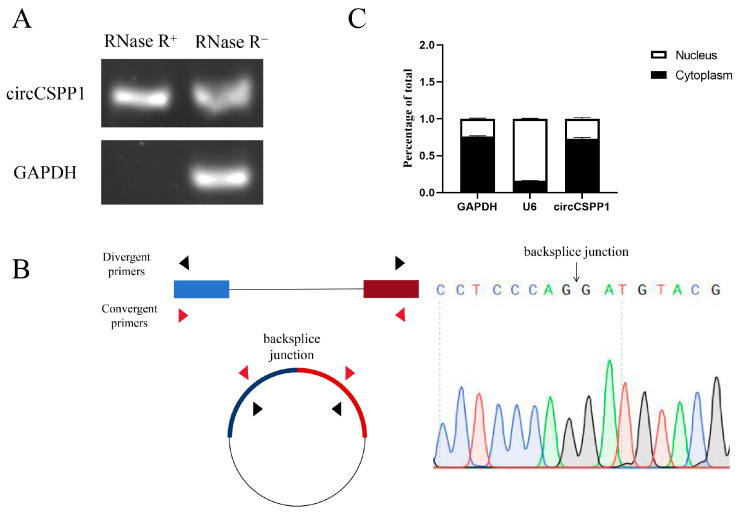
Identification and localisation of circCSPP1. (**A**) Amplification results of circCSPP1 after RNase R digestion. (**B**) The splicing junction sequencing diagram of circCSPP1. (**C**) Nucleoplasmic localisation of circCSPP1.

Cellular RNA was digested with RNase R enzyme, and the digested RNA and normal RNA were reverse transcribed into cDNA as a template for amplification of circCSPP1 and *GAPDH*, respectively. The results showed that both circCSPP1 and GAPDH were able to amplify the target fragment when normal RNA was used as a template, and when the RNA was digested with RNase R, circCSPP1 was able to amplify the target fragment, whereas *GAPDH* was not able to amplify the target fragment (Figure 1A). The PCR products were sequenced after agarose gel electrophoresis to determine the correct length of the target fragment, and the results showed that the circCSPP1 amplification products contained their own splice sites (Figure 1B). The above results indicated that circCSPP1 has a loop shape. ceRNA regulatory mechanisms are generally located in the cytoplasm, so nucleoplasmic localisation of circCSPP1 was performed, and the results revealed that circCSPP1 is mainly expressed in the cytoplasm (Figure 1C), suggesting that circCSPP1 has a possibility ceRNA-mediated regulation.

### 2.2. Verification of the Targeting Relationship Between circCSPP1 and miR-10a

**Figure 2:**
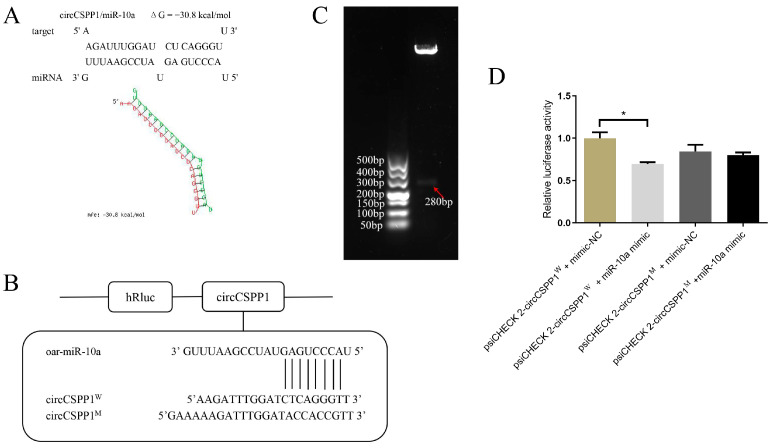
Validation of circCSPP1 with miR-10a targeting. (**A**) The prediction result of circCSPP1 with miR-10a. (**B**) The binding sites of circCSPP1 and miR-10a. (**C**) Validation of circCSPP1 amplification results. (**D**) Validation of circCSPP1 and miR-10a binding sites. * represents significant difference (*p* < 0.05).

Validation of the targeting relationship between miR-10a and *BMP7* was previously completed. In this study, binding site prediction of circCSPP1 and miR-10a was performed using RNAHybrid software version 2.0 [https://bibiserv.cebitec.uni-bielefeld.de/rnahybrid/ (accessed on 10 September 2020)], the presence of sequences complementarily paired with the seed sequence of miR-10a at 277–284 bp of circCSPP1 was found, and ΔG = −30.8 kcal/mol was predicted (Figure 2A). The circCSPP1 wild-type and mutant vectors were designed and constructed as shown in Figure 2B. These vectors were then co-transfected with miR-10a mimics/mimics-NC in 293T cells, allowing for the validation of the targeting relationship between circCSPP1 and miR-10a. The results showed that the fluorescence activity of the circCSPP1^W^ + miR-10a mimic group was significantly lower than that of the circCSPP1^W^ + miR-10a mimic-NC group (*p* < 0.05) and the fluorescence activity of the circCSPP1^M^ + miR-10a mimic group was not significantly different from that of the circCSPP1^M^ + miR-10a mimic-NC group (*p* > 0.05). There was no significant difference (*p* > 0.05) and the fluorescence activity was close to that of the circCSPP1^W^ + miR-10a mimic-NC group (Figure 2D), suggesting that circCSPP1 was able to bind to the sequence of the seed region of miR-10a in a targeting relationship.

### 2.3. circCSPP1 Competitively Binds miR-10a to Promote Proliferation of DPCs

**Figure 3:**
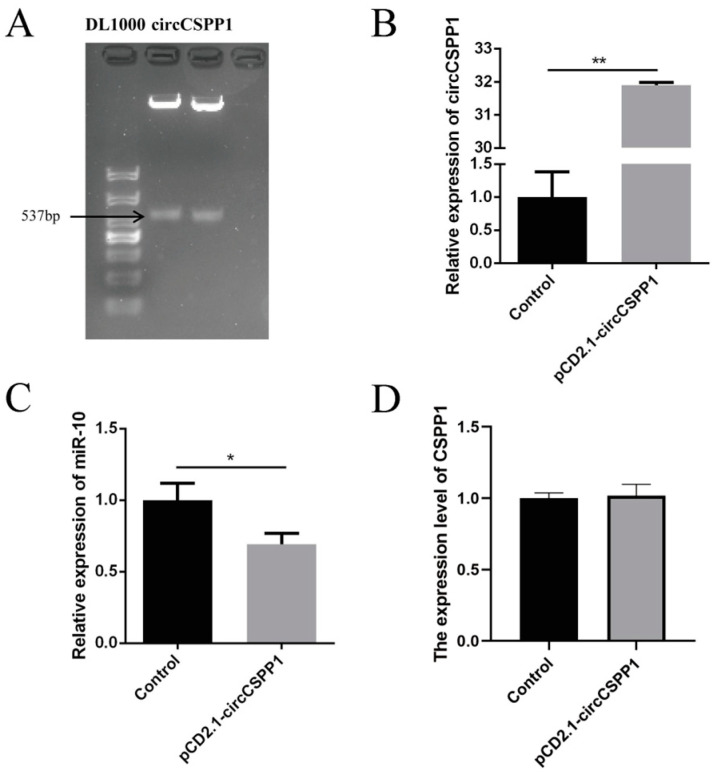
Validation of circCSPP1 full-length amplification and its transfection effect. (**A**) The full-length amplification results of circCSPP1. (**B**) The relative expression of circCSPP1. (**C**) The relative expression of miR-10a. (**D**) The relative expression of CSPP1. * represents significant difference (*p* < 0.05) and ** represents extremely significant difference (*p* < 0.01).

Based on the full-length sequence information of circCSPP1 in the sequencing results (Supplementary Table S1), a full-length 537 bp target fragment of circCSPP1 was obtained by PCR amplification (Figure 3A). Transfection of pCD2.1-circCSPP1 into Hu sheep DPCs showed that when circCSPP1 was overexpressed in DPCs, the expression of circCSPP1 was highly significantly increased compared to the control group (*p* < 0.01), suggesting that overexpression group was able to increase the expression level of circCSPP1 in dermal papilla cells (Figure 3B). However, despite this increase in circCSPP1 expression, it did not affect the expression level of CSPP1 (Figure 3D). Meanwhile, when circCSPP1 was overexpressed, the expression level of miR-10a was significantly lower than that of the control group (*p* < 0.05), suggesting that overexpression of circCSPP1 in the DPCs of Hu sheep was able to reduce the expression of miR-10a (Figure 3C).

**Figure 4:**
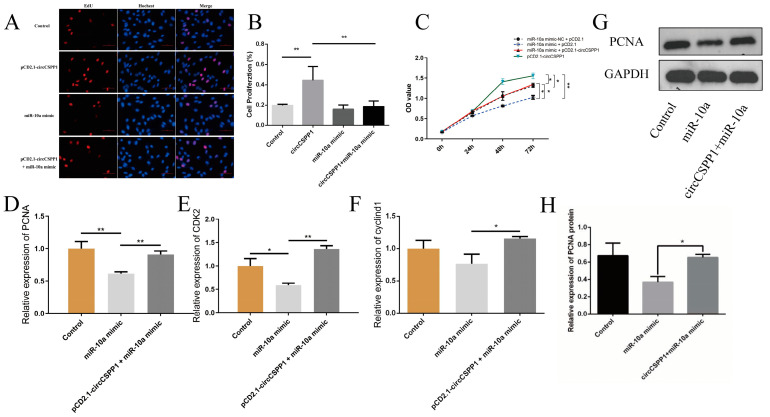
The effect of circCSPP1 targeting miR-10a on the proliferation of DPCs. (**A**) The effect of transfection with circCSPP1 overexpression vector after miR-10a overexpression on the proliferation of Hu sheep DPCs, the scale is 400 µm. (**B**) The rate of proliferating cells. (**C**) The viability of DPCs. (**D**) The relative mRNA expression of *PCNA*. (**E**) The relative mRNA expression of *CDK2*. (**F**) The relative mRNA expression of *cyclind1*. (**G**) The relative protein expression of PCNA. (**H**) The grayscale analysis of PCNA protein. * represents significant difference (*p* < 0.05) and ** represents extremely significant difference (*p* < 0.01).

To elucidate the impact of circCSPP1’s competitive binding to miR-10a on the proliferation of DPCs in Hu sheep, a rescue assay was used to investigate whether exogenous supplementation of circCSPP1 could counteract the inhibitory effect of miR-10a on DPCs. When circCSPP1 was overexpressed in DPCs, the number of proliferating cells was extremely significantly increased compared to the control group (*p* < 0.01). However, when miR-10a was overexpressed, the number of proliferating cells decreased compared to the control group. Nevertheless, when circCSPP1 was added during the overexpression of miR-10a, the number of proliferating cells was restored to that of the control group (Figure 4A,B). CCK-8 assay results showed that when miR-10a + pCD2.1-ciR was transfected into DPCs, their viability was significantly lower than that of the control group (*p* < 0.05). However, when miR-10a overexpression was accompanied by exogenous addition of circCSPP1, the viability of DPCs was restored to levels close to the control group. This suggests that exogenous circCSPP1 addition was capable of reversing the decrease in DPCs viability induced by miR-10a (Figure 4C). Analysis of the mRNA expression of *PCNA*, *CDK2*, and *cyclind1* in different experimental groups revealed that overexpression of miR-10a in DPCs resulted in a significant extremely significant decrease in the mRNA expression levels of *PCNA*, *CDK2*, and *cyclind1* (*p* < 0.05 or *p* < 0.01, respectively), and a corresponding decrease in PCNA protein expression levels was detected. When circCSPP1 is exogenously added, the mRNA expression levels of *PCNA*, *CDK2*, and *cyclind1* will return to the levels observed in the control group and the expression of PCNA protein will also recover (Figure 4D–H). The results indicate that circCSPP1 has the ability to promote the proliferation of DPCs in Hu sheep and the exogenous addition of circCSPP1 can reverse the decrease in cell proliferation caused by miR-10a, and also indicate that circCSPP1 can target miR-10a.

**Figure 5:**
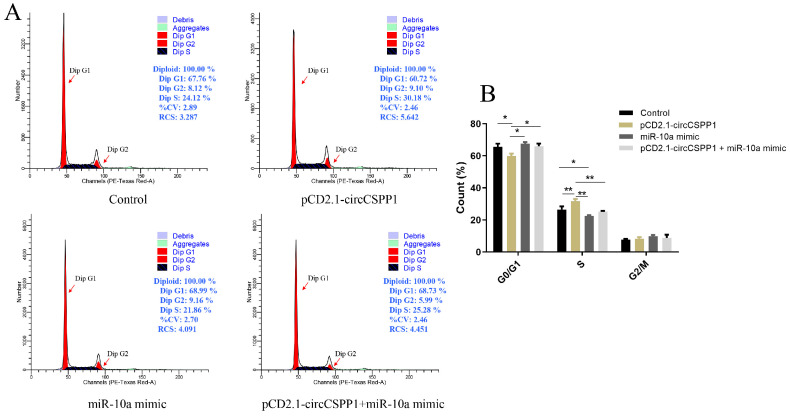
The effect of circCSPP1 targeting miR-10a on the cell cycle of DPCs. (**A**) Cell cycle. (**B**) The rate of proliferating cells. * represents significant difference (*p* < 0.05) and ** represents extremely significant difference (*p* < 0.01).

To clarify that circCSPP1 can competitively bind miR-10a to affect the cell cycle of DPCs in Hu sheep, the effect of exogenous addition of circCSPP1 on changes in cell cycle after overexpression of miR-10a was analysed using PI staining. After overexpression of circCSPP1, the S-phase of DPCs of Hu sheep was extremely significantly higher than that of the control group (*p* < 0.01), and after overexpression of miR-10a, the S-phase was decreased compared to that of the control group, but the S-phase of DPCs was restored to the level of the control group by the exogenous addition of circCSPP1 (Figure 5). The results showed that circCSPP1 had a promoting effect on the cell cycle of DPCs and that the exogenous addition of circCSPP1 is able to restore the slowed cell cycle progression by miR-10a overexpression.

### 2.4. The Effect of miR-10a on the Proliferation of DPCs

**Figure 6:**
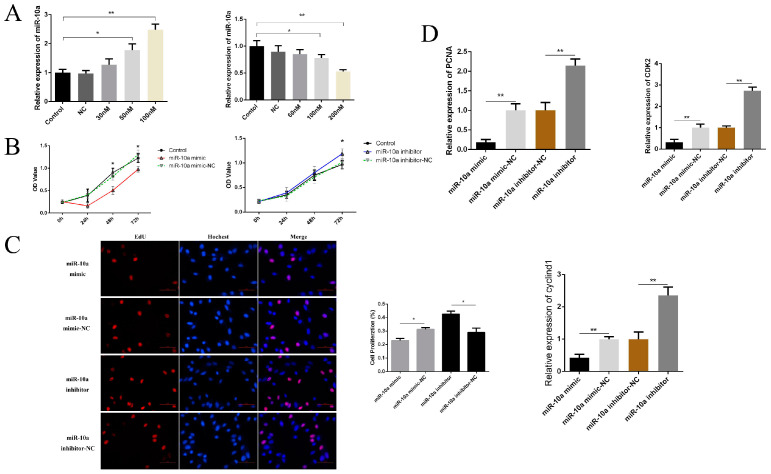
The effect of DPC proliferation after transfection of miR-10a mimic and inhibitor. (**A**) The transfection efficiency of miR-10a mimic and inhibitor under the different concentrations. (**B**) The viability of DPCs. (**C**) The effect of DPC proliferation after transfection of miR-10a mimic and inhibitor, the scale is 400 µm. (**D**) The relative mRNA expression of CDK2, PCNA, and cyclind1. * represents significant difference (*p* < 0.05) and ** represents extremely significant difference (*p* < 0.01).

The targeting relationship between miR-10a and *BMP7* has been previously verified [14]. The effect of miR-10a targeting *BMP7* on the proliferation of DPCs was further verified. Firstly, the expression of miR-10a was detected by transfecting different concentrations of miR-10a mimics and inhibitors into DPCs. The results showed that when the miR-10a mimic with a final concentration of 100 nM was transfected, the miR-10a expression level in the DPCs was extremely significantly higher than that in the mimic-NC group (*p* < 0.01). When miR-10a inhibitor with a final concentration of 200 nM was transfected, the miR-10a expression level in the DPCs was extremely significantly lower than that in the inhibitor-NC group (*p* < 0.01) (Figure 6A). Therefore, the final concentrations of 100 nM and 200 nM were ultimately chosen for the miR-10a mimic and inhibitor, respectively, as the transfection concentrations for subsequent procedures. The CCK-8 results showed that when miR-10a was overexpressed by transfecting the miR-10a mimic into DPCs, a significant decrease in cell viability was observed at 48 h and 72 h compared to the negative control group (*p* < 0.05). Conversely, when miR-10a expression was inhibited by transfecting miR-10a inhibitor, a significant increase trend at 72 h (*p* < 0.05) (Figure 6B). The EdU assay was performed to assess the proliferation of DPCs, and the results indicated that the amount of cell proliferation in the miR-10a mimic group was significantly lower than that in mimic-NC group (*p* < 0.05). Conversely, the amount of cell proliferation in the miR-10a inhibitor group was significantly higher than that in inhibitor-NC group (*p* < 0.05) (Figure 6C). These findings suggested that miR-10a exerted an inhibitory effect on the proliferation of DPCs.

Concurrently, the mRNA expression levels of genes related to cell proliferation and the cell cycle, namely *CDK2*, *PCNA*, and *cyclind1*, were examined after the overexpression and inhibition of miR-10a in DPCs. The results revealed that the mRNA expression level of *CDK2*, *PCNA*, and *cyclind1* genes was extremely significantly lower in the miR-10a mimic group than in the mimic-NC group (*p* < 0.01). Conversely, the mRNA expression level of these genes was extremely significantly higher in the miR-10a inhibitor group than in the inhibitor-NC group (*p* < 0.01) (Figure 6D).

### 2.5. The Effect of miR-10a Targeting BMP7 on the Proliferation of DPCs

**Figure 7:**
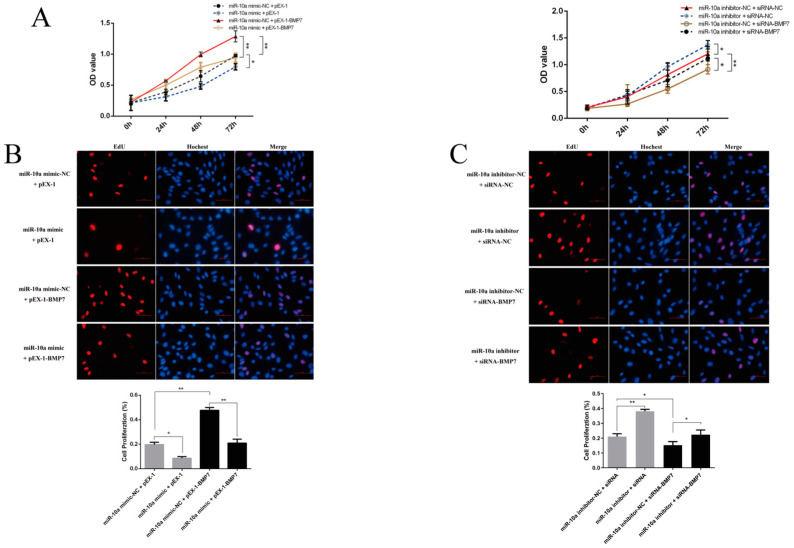
The effect of miR-10a targeting *BMP7* on the proliferation of DPCs. (**A**) The viability of DPCs. (**B**) The effect of exogenous addition of *BMP7* after miR-10a overexpression on the proliferation of DPCs, the scale is 400 µm. (**C**) The effect of exogenous addition of siRNA-*BMP7* after miR-10a inhibition on the proliferation of DPCs, the scale is 400 µm. * represents significant difference (*p* < 0.05) and ** represents extremely significant difference (*p* < 0.01).

When co-transfected with the miR-10a mimic and pEX-1, the viability/proliferation of DPCs was significantly lower than that of the control group (miR-10a mimic-NC + pEX-1) (*p* < 0.05), indicating that miR-10a can inhibit the cell viability and proliferation. When co-transfected with the miR-10a mimic-NC and pEX-1-*BMP7*, the viability/proliferation of DPCs was extremely significantly higher than that of the control group (*p* < 0.01), suggesting that *BMP7* can promote cell viability and proliferation. When pEX-1-*BMP7* was transfected after transfection with the miR-10a mimic, the cell viability/proliferation was found to be close to that of the control group, indicating that exogenously added *BMP7* can counteract the inhibitory effect of exogenously added miR-10a on DPCs (Figure 7A,B). Conversely, when co-transfected with miR-10a inhibitor and siRNA-NC, the cell viability was significantly higher than that of the control group (miR-10a inhibitor-NC + siRNA-NC) (*p* < 0.05) and the cell proliferation was extremely significantly higher than that of the control group (*p* < 0.01), indicating that the miR-10a inhibitor can promote cell viability and proliferation. When co-transfected with miR-10a inhibitor-NC and siRNA-*BMP7*, the cell viability/proliferation was significantly lower than that of the control group (*p* < 0.05), suggesting that siRNA-*BMP7* can inhibit cell viability and proliferation. When siRNA-*BMP7* was transfected after transfection with the miR-10a inhibitor, the cell viability/proliferation was found to be close to that of the control group, indicating that exogenously added siRNA-*BMP7* can counteract the promoting effect of exogenously added miR-10a inhibitor on the DPCs. The results demonstrated that miR-10a can target *BMP7* and inhibit the viability and proliferation of dermal papilla cells (Figure 7A,C).

## 3. Discussion

With the increasing research on hair curvature, the discovery of related genes has been increasing and research reports are growing. Our research teamhas previously shown that the *BMP7* gene is involved in the growth and development of hair follicles in the skin of Hu sheep, and it also affects the formation of wool curl and lambskin patterns. Wool growth is regulated by hair follicles, and the DPCs are the dermal part of the hair follicle, located at the base of the hair follicle and surrounded by hair matrix cells. As a signalling centre, DPCs send signals that act on the hair matrix cells and play an important role in their differentiation and hair growth [1]. The DPCs are the key cells for hair follicle growth and development [20] and play an important role in the process of hair curvature formation. Driskell et al. [21] found that *Sox*2+ DPCs produce straight hairs, whereas *Sox*2− DPCs produce curved zigzag hairs, and that the type of DPCs determines the hair curvature in mice. At the same time, Chi et al. [22] found that the number of DPCs was significantly correlated with the diameter and curvature of the hairs, and when the number of DPCs was reduced by specific knockdown method, the original straight hairs were changed to curved zigzag hairs. Therefore, it is thought that the number of DPCs affects the formation of wool curvature. The pattern of Hu sheep’s lambskin is caused by the curliness of wool. To study the pattern of lambskin, we first analysed the molecular mechanism from the perspective of wool curl formation. Dermal papilla cells are the core cells that influence hair curliness. Therefore, this study focuses on the proliferation of dermal papilla cells.

CircRNA is a type of non-coding RNA that lacks the 5′ cap and 3′ poly(A) tail structures, instead forming a closed circular RNA through covalent bonds [23]. It has become a research hotspot in modern animal husbandry. As circRNA research is still in its infancy, there are few circRNAs with known functions. Therefore, the excavation and identification of new circRNAs are the current research focus, and they are also the prerequisites for studying the functions and mechanisms of circRNAs. With the continuous development of high-throughput technology, more and more novel circRNAs have been identified in various tissues of domestic animals, and a large number of circRNAs show a stable structure, high abundance, and spatio-temporal specific expression. Existing studies have reported that circRNAs play important biological functions in various tissues of animals, including the foetal brain, mammary gland, pituitary gland, skeletal muscle, and intramuscular fat. The regulatory mechanisms underlying these functions are also gradually being discovered and validated [24]. CircRNAs function in a variety of ways, including as miRNA ‘sponges’ that competitively bind miRNAs to regulate gene expression. Previously, the differentially expressed non-coding RNAs and mRNAs between small wave and straight wool in Hu sheep lambskin were screened by RNA-seq, a ceRNA network including circCSPP1-miR-10a-*BMP7* was constructed, and the targeting relationship between miR-10a and *BMP7* was verified. Therefore, the present study focused on the competitive binding of circCSPP1 to miR-10a, which affects the proliferation of Hu sheep DPCs.

Since circRNAs are a class of tissue-specific and highly stably expressed cyclic RNAs, it is important to determine whether circCSPP1 is cyclic or not. In this experiment, the circular structure of circCSPP1 was confirmed through RNase R enzyme treatment and reverse primer PCR amplification. Wei and Li et al. [18,19,25] have studied the ceRNA regulatory network involved in bovine muscle proliferation and differentiation, utilizing the same methods to confirm the circular structure of circLMO7, circFGFR4, and circFUT10. These results are consistent with ours. At the same time, circLMO7, circFGFR4, and circFUT10 were primarily detected in the cytoplasm. Generally speaking, only circRNA primarily located in the cytoplasm may have a ceRNA regulatory mechanism. This study aims to verify the impact of the circCSPP1-miR-10a-*BMP7* ceRNA network on the DPC proliferation of Hu sheep. Therefore, further nuclear–cytoplasmic localization experiments were conducted to confirm that circCSPP1 is mainly distributed in the cytoplasm, suggesting the possibility of a ceRNA regulatory mechanism.

Regarding the extensive research on the regulatory network of ceRNAs, ciRS-7 stands out as the most thoroughly studied circRNA to date. CiRS-7 contains over 70 conserved binding sites for miR-7, enabling it to regulate the expression of numerous miR-7 target genes [26,27]. However, whether ciRS-7 inhibits or protects the expression of miR-7 is dependent on the cell type. Specifically, knocking out ciRS-7 in the mouse genome results in decreased expression levels of miR-7 [28], whereas other studies have observed a negative correlation between the expression of ciRS-7 and miR-7 [29]. CircBIRC6 and circCORO1C have been discovered to the functionality of human embryonic stem cells by acting as miRNA sponges to inhibit the miRNA-mediated suppression of pluripotency genes such as *NANOG*, *OCT4*, and *SOX2* [30]. CircZNF91 is induced during the differentiation of epidermal stem cells [31], and it has 24 binding sites for miR-23b-3p, which plays an important role in differentiation of keratinocytes [32]. In this study, the dual-luciferase reporter system was also employed to verify that circCSPP1 can bind to miR-10a. Combined with previous research showing that miR-10a can bind to *BMP7*, the circCSPP1-miR-10a-*BMP7* regulatory network was initially validated.

It is important to note that a circRNA may have both promotional and inhibitory effects. This is due to the fact that the majority of circRNA sequences possess multiple binding sites for different miRNAs. The oncogenic circCCDC66 contains multiple binding sites for miRNAs that target oncogenes, including miR-33b and miR-93, which specifically target the *MYC* oncogene [33]. In this study, circCSPP1 has been shown to promote the proliferation of DPCs by binding to miR-10a. However, it is important to note that when investigating the interactions of circCSPP1 with other miRNAs, there is a possibility that it may exhibit different functional roles. Indeed, the research on ceRNAs in the field of animal husbandry, particularly in relation to livestock production, has gradually increased in recent years. CircLMO7 regulates the expression of *HDAC4* by binding to miR-378a-3p, thereby promoting skeletal muscle differentiation and inhibiting cell proliferation [25,34]. CircSNX29 has been identified to competitively bind miR-744, thereby reversing the inhibitory effect of miR-774 on *Wnt5a*, activating the Wnt5a/Ca^2+^ signalling pathway and promoting the differentiation of skeletal muscle cells while inhibiting cell proliferation [35]. CircTTN promotes the proliferation and differentiation of bovine primary myoblasts by competitively binding to miR-432 and activating the IGF2/PI3K/AKT signalling pathway [36]. Yin et al. [37] identified a key circRNA-1926 in the secondary hair follicles of cashmere goats during the growth and regression phases. They then verified that circRNA-1926 positively regulates the expression of *CDK19* by competitively binding to miR-148a/b-3p, thereby promoting the differentiation of secondary hair follicle stem cells into hair follicles in cashmere goats. All the above studies are based on the ceRNA regulatory mechanism, where circRNA competitively binds to miRNA to positively regulate gene expression, thereby affecting cell proliferation and differentiation. Therefore, based on the established findings that circCSPP1 targets miR-10a and miR-10a targets *BMP7*, this study further validated through rescue experiments that circCSPP1 can competitively bind to miR-10a to positively regulate the expression of *BMP7*, thereby promoting the proliferation of dermal papilla cells in Hu sheep.

## 4. Materials and Methods

### 4.1. Samples and Ethics Statement

The DPCs were obtained from laboratory preservation, and skin tissue samples were collected from 3-day-old Hu lamb in Suzhou Sheep Farm (Suzhou, Jiangsu, China). The experiment operation is approved by the Animal Ethics Committee of Yangzhou University (approval number: No. 202103279).

### 4.2. Identification of circCSPP1

Based on the splicing site position of circCSPP1, divergent primers were designed (Table 1). Total RNA was extracted from DPCs of Hu sheep and subsequently digested with the RNase R enzyme. Using both the RNA before and after digestion as templates, the fragments of circCSPP1 and *GAPDH* were amplified. The PCR products were subsequently detected through agarose gel electrophoresis and then sent to Beijing Tsingke Biotech Co., Ltd., Beijing, China for sequencing to verify sequence accuracy.

### 4.3. Cell Culture and Transfection

The DPCs and 293T used in this experiment were preserved in our laboratory. The cell culture condition was 10% foetal bovine serum (FBS) (Gibco, Grand Island, NY, USA), DMEM/F12 (HyClone, Logan, UT, USA), and 1% penicillin-streptomycin (Solarbio, Beijing, China). According to the experimental requirement, the cells were cultured in petri dishes of various sizes at 37 °C with 5% CO_2_. The cell transfection was performed using jetPRIME transfection reagent (Polyplus, Illkirch, France).

### 4.4. Plasmids Construction, RNA Oligonucleotides

Based on the sequence information of circCSPP1 (Supplementary Table S1), the potential target binding sites of circCSPP1 and miR-10a were predicted using the RNAhybrid software version 2.0 [https://bibiserv.cebitec.uni-bielefeld.de/rnahybrid/ (accessed on 10 September 2020)], and primers were designed to include miR-10a binding site, as well as restriction enzyme sites for Xho I (5′C^TCGAG 3′) and Not I (5′GC^GGCCGC3′). The RNA from the hair follicles of lambskin was reverse transcribed into cDNA, and then the circCSPP1 sequence containing the miR-10a binding site was obtained by PCR amplification and ligated into the psiCHECK-2 vector to construct the wild-type vector circCSPP1^W^. The wild-type vector was used as a template to construct the mutant-type vector circCSPP1^M^ using the Fast Site-Directed Mutagenesis Kit (Tiangen, Beijing, China). Primers were designed for the full-length circCSPP1 sequence, and Kpn I (5′ GGTAC^C 3′) and BamH I (5′ G^GATCC 3′) were used as enzyme cleavage sites to construct the pCD2.1-circCSPP1 vector. The primers’ information is in Table 2. The overexpression vector pEX-1-*BMP7* and the interfering sequences siRNA-*BMP7* were prepared in previous study [8].

### 4.5. Dual Luciferase Reporter Gene Assay

The 293T cells were used to perform cell recovery and culture in a 24-well plate. Transfection was carried out when the cells had grown to about 70%. The transfection groups were circCSPP1^W^ + miR-10a mimic-NC, circCSPP1^W^ + miR-10a mimic, circCSPP1^M^ + miR-10a mimic-NC, and circCSPP1^M^ + miR-10a mimic. Each group was set up with three replicate groups. Cells were allowed to transfect for 24 h and fluorescence detection was performed using the Dual-Luciferase Report Kit (Vazyme, Nanjing, China).

### 4.6. RT-qPCR

According to the universal principles of miRNA primer design, the upstream primer was designed for miR-10a, the downstream primer was the universal primer provided with the miRcute Enhanced Fluorescence Quantification Kit (Tiangen, Beijing, China), and U6 was used as a housekeeping reference with a Tm of around 65 °C (Table 3). According to the instructions of the miRNA reverse transcription kit (poly(A) tailing method) (Tiangen, Beijing, China), cDNA first strand synthesis was performed on cellular RNA, and using cDNA as a template, quantitative detection was carried out using the miRcute Enhanced Fluorescent Quantitative Kit (Tiangen, Beijing, China).

The mRNA relative expression levels of proliferation marker genes *CDK2* (FJ422550.1), *PCNA* (XM_004014340.4), *cyclind1* (XM_027959928.1), *BMP7* (KF925831), and *CSPP1*(XM_060393758.1) were determined using *GAPDH* (NM_001190390.1) as a housekeeping reference (Table 4). The first strand of cDNA was synthesised using the FastKing cDNA one-step reverse transcription kit (Tiangen, Beijing, China) and the SYBR^®^ Premix Ex Taq II Reagent Kit (Takara, Dalian, China) was used to detect mRNA relative expression.

### 4.7. Cell Proliferation

After the groups were transfected for 24 h, the cells were digested with 0.25% trypsin. Then, the cell suspension was prepared by adding the appropriate complete medium and dispensed into 96-well plates (100 µL/well). The CCK-8 Kit (Vazyme, Nanjing, China) was used to detect cell viability at 24 h (after transfection), 48 h, 72 h, and 48 h. The OD value was determined at 450 nm using a microplate reader (EnSpire, Perkin Elmer, Waltham, MA, USA). Cell samples were processed using the EdU Apollo In Vitro Imaging Kit (RiboBio, Guangzhou, China), imaged using an inverted fluorescence microscope (Nikon, Tokyo, Japan), and analysed using Image Pro software version 7.1.

### 4.8. Cell Cycle

After transfection for 24 h, the medium was replaced with fresh medium and the cells were further cultured for 24–48 h. When the cell density reached 80%, the cell cycle was detected using the Cell Cycle and Apoptosis Analysis Kit (Beyotime, Shanghai, China).

### 4.9. Western Blot

RIPA lysates (Beyotime, Shanghai, China) were used to lyse cells and collect proteins. Protein denaturation was performed according to the protein concentration determined using the Enhanced BCA Protein Assay Kit (Beyotime, Shanghai, China). After SDS-PAGE electrophoresis, the proteins were transferred to a PVDF membrane. The primary antibodies were BMP7 (Customised by Hangzhou Hua’an Biotechnology Co., Ltd., Hangzhou, China, 1:2000), PCNA (Jingjie, Hangzhou, China, 1:2500), and GAPDH (proteintech, Wuhan, China, 1:5000). The secondary antibodies were HRP-conjugated Goat anti-Mouse IgG (H&L) (ABclonal, Wuhan, China, 1:5000). Protein visualization was performed using the ECL Western Blot Kit (BioSharp, Hefei, China).

### 4.10. Statistical Analysis

The relative expression was calculated by the 2^−ΔΔCT^ method [38]. The SPSS 13.0 software was used to perform the independent samples *t*-test (* represents significant difference, *p* < 0.05, ** represents extremely significant difference, *p* < 0.01). GraphPad Prism 6 software was used for drawing. Three biological replicates were used for each analysis and results are expressed as mean ± SEM.

## 5. Conclusions

**Figure 8:**
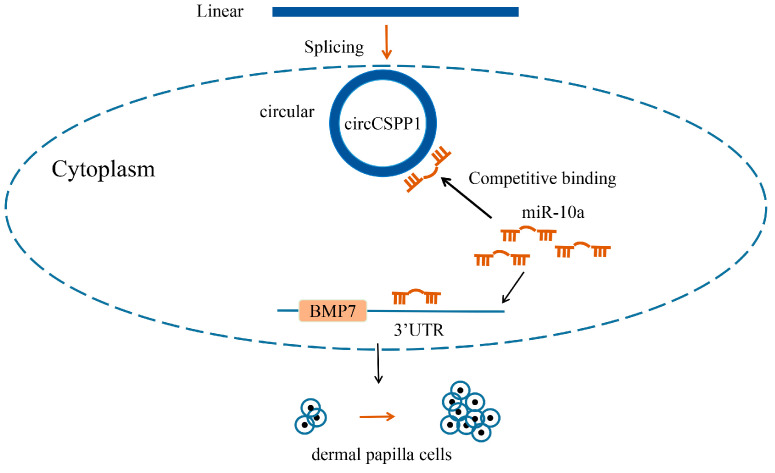
Model diagram of ceRNA regulatory network.

In conclusion, we reveal that circCSPP1 is capable of regulating the expression of *BMP7* binding to miR-10a, thereby promoting the proliferation of DPCs in Hu sheep. Our study will provide benefits for further research into the processes of the growth and development of hair follicles (Figure 8).

## Figures and Tables

**Table 1:** The PCR amplification primers’ information.

Name	Primer Sequence (5′-3′)	Product Length (bp)
circCSPP1	F: GTCTGCCCCATCTGTCCCA	280
	R: CACCCCAAAGAGCATTCCC	
*GAPDH*	F: GTCGGAGTGAACGGATTTGG	196
	R: CATTGATGACGAGCTTCCCG	

**Table 2:** The primer information of circCSPP1.

Name	Primers (5′-3′)	Product Length (bp)
circCSPP1^W^	F: ccgCTCGAGACCTACTTATCGAGAGACGTGC R: atttGCGGCCGCACTCTGGGTCTCTCAGGTGG	280
circCSPP1^M^	F: GAAAAAGATTTGGAT**ACCACCGT**TGCAGCTTCTGGAGC R: GAAGCTGCA**ACGGTGGT**ATCCAAATCTTTTTCATTAC	280
circCSPP1	F: ggGGTACCGATGTACGGGAACAGACGAGG R: cgGGATCCCTGGGAGGCACCATGTCA	537

Note: Lowercase represents protected bases, and underline represents the enzyme cut site. Bold represents target binding site mutation sequences.

**Table 3:** The primers information of miR-10a.

Name	Forward Primers (5′-3′)	Tm (°C)
miR-10a	CGATACCCTGTAGATCCGAATTTG	65
U6	TGGAACGTATCAGAGAAGATTAGCA	

**Table 4:** The primers information of genes.

Name	Primers (5′-3′)	Product Length (bp)
*CDK2*	F: TGGGCCAGGCAGGATTTTAG R: GTCGAAGGTGAGGTACTGGC	166
*PCNA*	F: TCTGCAAGTGGAGAACTTGGAA R: AGGAGACAGTGGAGTGGCTT	162
*cyclind1*	F: GCTTCCTCTCCTATCACCGC R: GGCTTTGGGGTCCAAGTTCT	149
*BMP7*	F: TGAGTTCCGCATTTACAAGG R: GTGGCTGTGATGTCAAAAAC	177
*CSPP1*	F: TGAGGATCGTGCTTTTGATAAAC R: AGGAGTTCGGTAGTTCGCAG	193
*GAPDH*	F: GTCGGAGTGAACGGATTTGG R: CATTGATGACGAGCTTCCCG	196

## Data Availability

All the data of this study are presented in the manuscript.

## Supplementary Materials

The supporting information can be downloaded at: https://www.mdpi.com/article/10.3390/ijms252111547/s1.
